# Relating Instructional Design Components to the Effectiveness of Internet-Based Mindfulness Interventions: A Critical Interpretive Synthesis

**DOI:** 10.2196/12497

**Published:** 2019-11-27

**Authors:** Marie Lippmann, Helena Laudel, Marlene Heinzle, Susanne Narciss

**Affiliations:** 1 Department of Psychology California State University Chico, CA United States; 2 Psychology of Learning and Instruction Faculty of Psychology - School of Science Technische Universität Dresden Dresden Germany; 3 Department of Media, Cognition and Communication Faculty of Humanities University of Copenhagen Copenhagen Denmark

**Keywords:** mindfulness, internet, instructional design

## Abstract

**Background:**

Internet-based mindfulness interventions are a promising approach to address challenges in the dissemination and implementation of mindfulness interventions, but it is unclear how the instructional design components of such interventions are associated with intervention effectiveness.

**Objective:**

The objective of this study was to identify the instructional design components of the internet-based mindfulness interventions and provide a framework for the classification of those components relative to the intervention effectiveness.

**Methods:**

The critical interpretive synthesis method was applied. In phase 1, a strategic literature review was conducted to generate hypotheses for the relationship between the effectiveness of internet-based mindfulness interventions and the instructional design components of those interventions. In phase 2, the literature review was extended to systematically explore and revise the hypotheses from phase 1.

**Results:**

A total of 18 studies were identified in phase 1; 14 additional studies were identified in phase 2. Of the 32 internet-based mindfulness interventions, 18 were classified as more effective, 11 as less effective, and only 3 as ineffective. The effectiveness of the interventions increased with the level of support provided by the instructional design components. The main difference between effective and ineffective interventions was the presence of just-in-time information in the form of reminders. More effective interventions included more supportive information (scores: 1.91 in phases 1 and 2) than less effective interventions (scores: 1.00 in phase 1 and 1.80 in phase 2), more part-task practice (scores: 1.18 in phase 1 and 1.60 in phase 2) than less effective interventions (scores: 0.33 in phase 1 and 1.40 in phase 2), and provided more just-in-time information (scores: 1.35 in phase 1 and 1.67 in phase 2) than less effective interventions (scores: 0.83 in phase 1 and 1.60 in phase 2). The average duration of more effective, less effective, and ineffective interventions differed for the studies of phase 1, with more effective interventions taking up more time (7.45 weeks) than less effective (4.58 weeks) or ineffective interventions (3 weeks). However, this difference did not extend to the studies of phase 2, with comparable average durations of effective (5.86 weeks), less effective (5.6 weeks), and ineffective (7 weeks) interventions.

**Conclusions:**

Our results suggest that to be effective, internet-based mindfulness interventions must contain 4 instructional design components: formal learning tasks, supportive information, part-task practice, and just-in-time information. The effectiveness of the interventions increases with the level of support provided by each of these instructional design components.

## Introduction

### Background

Many medical conditions are accompanied by experiences of discomfort, worry, rumination, and anxiety [1,2]. The practice of mindfulness helps individuals who suffer from medical and psychological conditions by decreasing the perceived effects of their symptoms and increasing psychological well-being [3,4]. Central to the concept of mindfulness is the idea of cultivating a nonjudgmental and accepting awareness of present experiences (ie, thoughts, feelings, and bodily sensations) as they arise [5]. As mindfulness is a complex skill that requires learning and practice, mindfulness-based interventions have been developed and implemented to assist individuals in mastering this skill [6,7]. Mindfulness-based interventions are typically administered in person but face challenges in terms of their dissemination and implementation. Internet-based mindfulness interventions are a promising new approach to address these challenges [8]. Several recent reviews provide insights into the effectiveness of internet-based mindfulness interventions for a variety of outcome measures [9-15]. However, no research has yet investigated how the design of those interventions is associated with intervention effectiveness. This study closes this gap in the literature by identifying instructional design components of internet-based mindfulness interventions and providing a framework for the classification of those components, relative to the intervention effectiveness.

### Internet-Based Mindfulness Interventions

Internet-based mindfulness interventions, as a subcategory of internet-based health interventions, have the potential to reach a large number of potential users, extend intervention accessibility to individuals with economic and transportation restrictions, increase intervention convenience through greater flexibility in use and application, avoid social stigma of therapeutic settings, and increase cost-effectiveness for both providers and clients [16-18]. In the past 5 years, 7 reviews have systematically investigated the effectiveness of internet-based mindfulness interventions.

Spijkerman and Bohlmeijer [9] examined 15 randomized controlled trials, comparing internet-based mindfulness interventions with control conditions. They found the internet-based mindfulness interventions to have significant small-to-moderate effects on mental health.

Fish et al [10] reviewed 10 technology-based mindfulness interventions aimed at clinical outcomes of mental health (stress, depression, and anxiety) and found that 8 studies produced significant effects but with varying effect sizes. The authors point out that they found it difficult to draw conclusions about intervention effectiveness relative to design components of the interventions, such as construction, length, and delivery, and explicitly call for further research to investigate this issue [10].

Toivonen et al [11] reviewed 16 internet-based mindfulness interventions aimed at physiological symptoms (eg, cancer, chronic pain or fibromyalgia, irritable bowel syndrome, epilepsy, heart disease, tinnitus, and acquired brain injury). They found that the majority of the studies reported positive effects of the internet-based mindfulness interventions compared with traditional treatment on a multitude of outcomes, including pain acceptance, coping mechanisms, and symptoms of depression [11]. The authors found mixed results when comparing internet-based mindfulness interventions to active control groups receiving, for example, cognitive behavioral therapy [11].

Heber et al [12] reviewed the effectiveness of internet- and computer-based stress management interventions in a meta-analysis including 26 comparisons. The authors found large effect sizes for the investigated interventions, relative to control groups, in terms of stress reduction; small effects were obtained for depression [12]. Subgroup analyses revealed that guided interventions were more effective than unguided interventions, and the authors found differences in intervention effectiveness based on the design characteristics of duration and intervention content [12], thereby highlighting the need for more research on the design of internet-based interventions and potential relationships between design and intervention effectiveness.

Lyzwinski et al [13] reviewed 21 internet-based mindfulness interventions for stress, maladaptive weight-related behaviors, and weight loss. They found that most interventions were effective for stress reduction. Conclusions about intervention effectiveness for weight-related behaviors could not be drawn because not enough studies with weight-related outcomes were identified [13].

Mikolasek et al [14] reviewed 17 empirical studies on internet-based mindfulness or relaxation interventions for medical conditions (eg, irritable bowel syndrome, cancer, chronic pain, surgery, and hypertension). This review found that the internet-based mindfulness or relaxation interventions were mostly effective, with varying effect sizes, but it found no effects for stress [14]. In the discussion of their findings, the authors point to differences in intervention design, such as intervention dose and regularity, as potential sources for the heterogeneity in intervention effectiveness [14].

Finally, Sevilla-Llewellyn-Jones et al [15] reviewed 12 internet-based mindfulness interventions for mental health in clinical populations and found that the internet-based mindfulness interventions were effective in reducing depression and anxiety while enhancing the quality of life and mindfulness skills, particularly in individuals with clinical anxiety. The authors point to challenges in the interpretation of the results based on the heterogeneity of the interventions and their components [15], providing further incentive to investigate the design of internet-based mindfulness interventions.

Overall, the reviews show heterogeneous, but predominantly encouraging, results in support of the effectiveness of internet-based mindfulness interventions aimed at a variety of mental and physical health conditions [9-15]. The authors of the majority of those reviews point out that the extent to which such findings can be generalized is limited by the large variety in components of internet-based interventions, including differences in content, scheduling, guidance, and support [10,12,14,15]. As a result, it is unclear which design components are associated with intervention effectiveness, and more research is needed to investigate the design of internet-based mindfulness interventions relative to their effectiveness.

### Relevant Components of Internet-Based Mindfulness Interventions: Instructional Design Perspective

Employing instructional design process models increases learning outcomes across a variety of contexts [19]. The need for instructional design in developing internet-based interventions is based on the premise that technology components are more likely to have positive effects on learning processes and outcomes when intervention designers take a learner-centered and need-based approach [19]. As mindfulness can be viewed as a complex skill demanding extensive amounts of practice to be learned and mastered [5], instructional design models provide powerful tools to identify relevant design components. An instructional design model that is particularly suitable to apply to complex learning processes, such as establishing and mastering mindfulness, is the 4-component instructional design (4C/ID) model [20]. The 4C/ID model comprises 4 core components.

The first component comprises *learning tasks* (LTs) that are authentic whole-task experiences. In the context of internet-based mindfulness interventions, LTs are represented by formal mindfulness exercises, typically guided meditations in the format of audio files. LTs are further specified in terms of their content and scheduling and whether they are tailored to specific conditions or target specific populations [21,22]. All of these aspects of LTs are relevant to intervention effects [21-23] and are, therefore, considered in this review.

The second component in the 4C/ID model refers to *supportive information* (SI) assisting learners in the acquisition and performance of nonrecurrent aspects of the LT to help establish correct mental models and appropriate cognitive strategies. In the context of internet-based mindfulness interventions, SI is represented by reflection exercises, psychoeducative information, and peer support in forums or chat rooms. SI seems to facilitate intervention effects for internet-based health interventions, but the effects are difficult to gauge because the SI is typically presented in addition to the main intervention [24].

The third component in the 4C/ID model refers to *just-in-time information* (JIT) that concerns information supporting the performance of recurrent aspects of the LT.

In terms of self-help programs, Cavanagh et al [25] found larger effects for programs with guiding prompts than for unguided programs. In internet-based mindfulness interventions, JIT is represented by prompts and reminders encouraging continuous, regular practice.

The fourth component in the 4C/ID model comprises *part-task practice* (PTP) that refers to the additional practice of selected recurrent skills that demand a certain level of automation. In internet-based mindfulness interventions, PTP is represented by informal practice exercises aimed at practicing the established mindfulness skills during everyday activities, such as mindful eating or walking.

**Figure 1:**
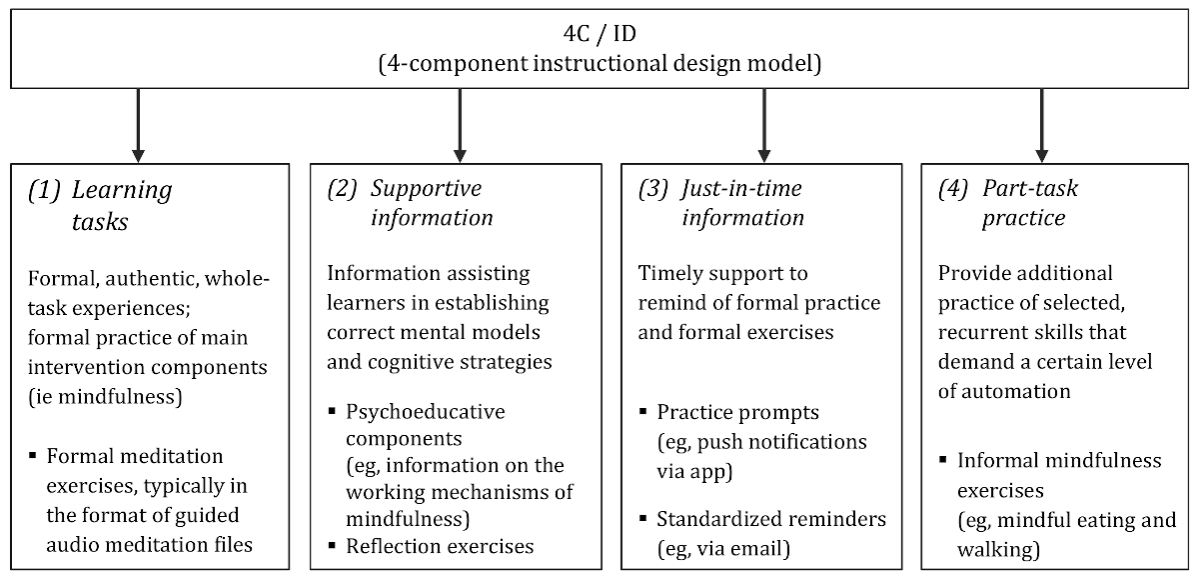
Four-component instructional design model.

In face-to-face interventions, both formal and informal practices have been recognized to play a key role in the development of mindfulness [3] and are, therefore, considered relevant to this review. For an overview of the components of internet-based mindfulness interventions mapped onto the components of the 4C/ID model, see Figure 1.

### Research Questions

With reference to the 4C/ID model, we applied the critical interpretive synthesis (CIS) method [26] to address the following 2 research questions:

1. *Which instructional design components can be identified in existing internet-based mindfulness interventions?*
2. *How can these design components be classified relative to the intervention effectiveness?*

## Methods

### The Critical Interpretive Synthesis Method

A typical approach to answering research questions from the existing literature is the Cochrane-style systematic review [26]. The CIS method provides an alternative to systematic reviews whenever the literature does not provide a sufficient foundation for a meta-analysis. As thus far, no empirical studies have investigated the instructional design of internet-based mindfulness interventions, we utilized strategic elements of systematic reviews but implemented those within the more flexible CIS method [21]. In contrast to systematic reviews, CIS does not rely on exhaustive literature searches, rigid inclusion criteria, and quality assessments but employs techniques from qualitative research, such as diversity sampling, to generate hypotheses and systematically explore those in an iterative and dynamic review process [26]. To address our research questions, we implemented CIS in 2 phases.

### Phase 1: Diversity Sampling and Generation of Hypotheses

#### Aim

The aim in phase 1 was to obtain a diverse sample of the existing literature on internet-based mindfulness interventions to identify instructional design components and generate a framework for classifying the effectiveness of the interventions relative to their design components.

#### Inclusion and Exclusion Criteria

With regard to population, we applied no restriction criteria because the transdiagnostic applicability of mindfulness is likely to result in a great variability of outcomes for varying populations, and we were interested in obtaining a diverse sample of the literature. With regard to the inclusion criteria for the intervention, we defined that interventions had to be delivered through the internet (ie, via a website or a mobile phone app) and contain formal mindfulness exercises as their main component to ensure the comparability of LTs across studies. In consequence, studies employing multicomponent interventions such as the Acceptance and Commitment Therapy [26] were not considered. Furthermore, interventions had to be delivered asynchronously, excluding interventions with live delivery, for example, via videoconferencing, to ensure the comparability of guidance and support components across studies. Only studies with control groups (typically waitlist controls) were considered. The intervention outcomes had to be indicative of mental or physical health. Relevant empirical studies had to have been published in an international peer-reviewed journal in English language. As both mindfulness- and internet-based interventions have predominantly emerged within the past two decades, no time restrictions were applied.

#### Search Strategy

A literature search was conducted in the databases PsycINFO, PsycARTICLES, PubMed, and Web of Science between February 2016 and October 2018.

After conducting a set of preliminary searches to identify the most accurate key words, the following search terms were included in all 4 databases (*mindfulness OR *mindful OR *meditation) AND (*internet OR *web OR *online OR *smartphone OR *app OR *mobile). The titles and abstracts of all search results were first screened for relevance. After removing duplicates of the publications identified as relevant, unavailable results were requested from the authors. On the basis of the results identified as dissertations, an additional author search was conducted to determine if the reported trials had been published in the meantime. All remaining studies were then subjected to a full-text screening. In the last step, the references of the included studies were screened to identify relevant research not covered by the database searches.

#### Quality Assessment in Terms of Risk Bias

Risk bias was assessed with the guidelines developed by the Cochrane Back Review Group [27] that had been successfully employed in a previous review on mindfulness-based interventions [28]. Assessment criteria focused on whether (1) methods of randomization were reported, (2) intervention outcomes were assessed with standardized measures, (3) a follow-up assessment was performed, (4) analyses included an intention-to-treat analysis, (5) sample characteristics were reported, (6) characteristics of withdrawals and dropouts were reported, and (7) studies contained detailed intervention descriptions. For each of the 7 criteria, 2 points were awarded if the criterion was reported and adhered to, 1 point if the criterion was reported but not adhered to, and 0 points if the criterion was not reported. The sum of the awarded points serves as an indicator of study quality, with 0 to 7 points indicating low, 8 to 11 indicating moderate, and 12 to 14 indicating high quality.

#### Review Strategy

For each included study, general information including authors, publication year, and country was recorded. The quality of the included studies was assessed in terms of risk bias. Sample characteristics including gender, age, and medical indications were retrieved. Outcome measures and characteristics of the control group were recorded. Group differences were assessed in terms of between- and within-group effects. The ranges of the effect sizes for the main outcome measures were recorded whenever effect sizes were reported in the original studies. Whenever effect sizes were not reported, we recorded reported *P* values instead. The effectiveness of the interventions was assessed with the criteria for defining intervention effectiveness [21]. The operationalization of those criteria is shown in Table 1.

The instructional design components of each intervention were identified and mapped onto the 4 components of the 4C/ID model (ie, LTs, SI, JIT, and PTP). In addition, information on the duration and scheduling of the interventions were recorded, and reports of adherence and acceptance were included whenever they were reported in the original studies.

**Table 1:** Criteria for defining intervention effectiveness according to Morrison et al (2012).

Intervention code	Criteria
More effective	The intervention led to improvement on majority of outcomes measures. The intervention was at least as effective as comparison groups. The intervention was more effective than waiting list or no intervention control groups.
Less effective	The intervention led to improvement on minority of outcomes measures. The intervention was not necessarily as effective as comparison groups. The intervention was more effective than waiting list or no intervention control groups.
Ineffective	The intervention did not lead to improvement on any of the outcome measures. The intervention was no more effective than waiting list or no intervention control groups.

#### Generation of Hypotheses for Phase 2

To systematically evaluate the instructional design components of the interventions relative to the intervention effectiveness, we constructed and implemented the following scoring system: each intervention received points in the range from 0 to 2 for each of the 4C/ID components. The operationalization of those points relative to the 4C/ID components is reported in Table 2.

Each intervention was scored according to this system. In a next step, average scores were computed for each of the 4C/ID components and mapped onto intervention effectiveness. These rating processes were conducted by two independent raters, and interrater reliability was computed.

On the basis of the review of this first set of studies, we systematically generated hypotheses regarding the association between instructional design components of internet-based mindfulness interventions and intervention effectiveness. According to the CIS procedure [26], a second literature search (representative sampling) was then conducted to explore whether the hypotheses from phase 1 were generalizable and consistent.

**Table 2:** Intervention ratings for 4-component instructional design components (duration was 1 point per week, and in case of varying data count, the duration was longest).

Score (points)	Learning task	Supportive information	Part-task practice	Just-in-time information
0	Not existent	Not existent	Not existent	Not existent
1	Existent but not described; formal exercises implemented less than twice per week; formal exercises stable in content	Existent but not described; educational material provided only once; optional contact in case of questions or problems	Existent but not described; 1 informal exercise on a single distinct topic; exercises only once per week or less	Existent but not described; reminders once per week or less; reminders only when adherence was absent
2	Described formal exercises with varying content; implemented at least twice per week	Continuously accessible educational, supportive material; reflection exercises (eg, as diary or log writing)	Several unstructured informal exercises across a variety of topics; implemented at least twice per week	1 reminder ahead of each scheduled practice; adjustable reminders; prompts with monitoring information

### Phase 2: Representative Sampling

#### Aim

The aim of phase 2 was to explore the extent to which the hypotheses from phase 1 were consistent and generalizable across an additional set of empirical studies.

#### Inclusion and Exclusion Criteria

Inclusion and exclusion criteria were identical to those in phase 1.

#### Search Strategy

To identify a representative sample of studies that may include more recent research, a systematic literature search was conducted to identify systematic reviews on the topic of internet-based mindfulness interventions published over the last 4 years. The studies reported in those reviews were identified, inclusion and exclusion criteria were applied, and the remaining studies were compared with the studies from the search in phase 1. This comparison revealed that all studies from the reviews matching our inclusion criteria were already represented in our search in phase 1. Therefore, a hand search in JMIR-relevant journals of the past 3 years was conducted, and the databases PubMed, PsycARTICLES, and PsycINFO were searched again for more recent studies in the years 2017 to 2019. For this hand search, the search string from phase 1 was used, titles and abstracts were scanned for relevance, duplicates were removed, and the remaining articles were subjected to a full-text screening.

#### Review Strategy

The review strategy from phase 1 was applied again, with the goal of identifying any instructional design components that might have not occurred in phase 1. Then, the associations between instructional design components and intervention effectiveness were examined.

## Results

### Phase 1: Diversity Sampling and Generation of Hypotheses

**Figure 2:**
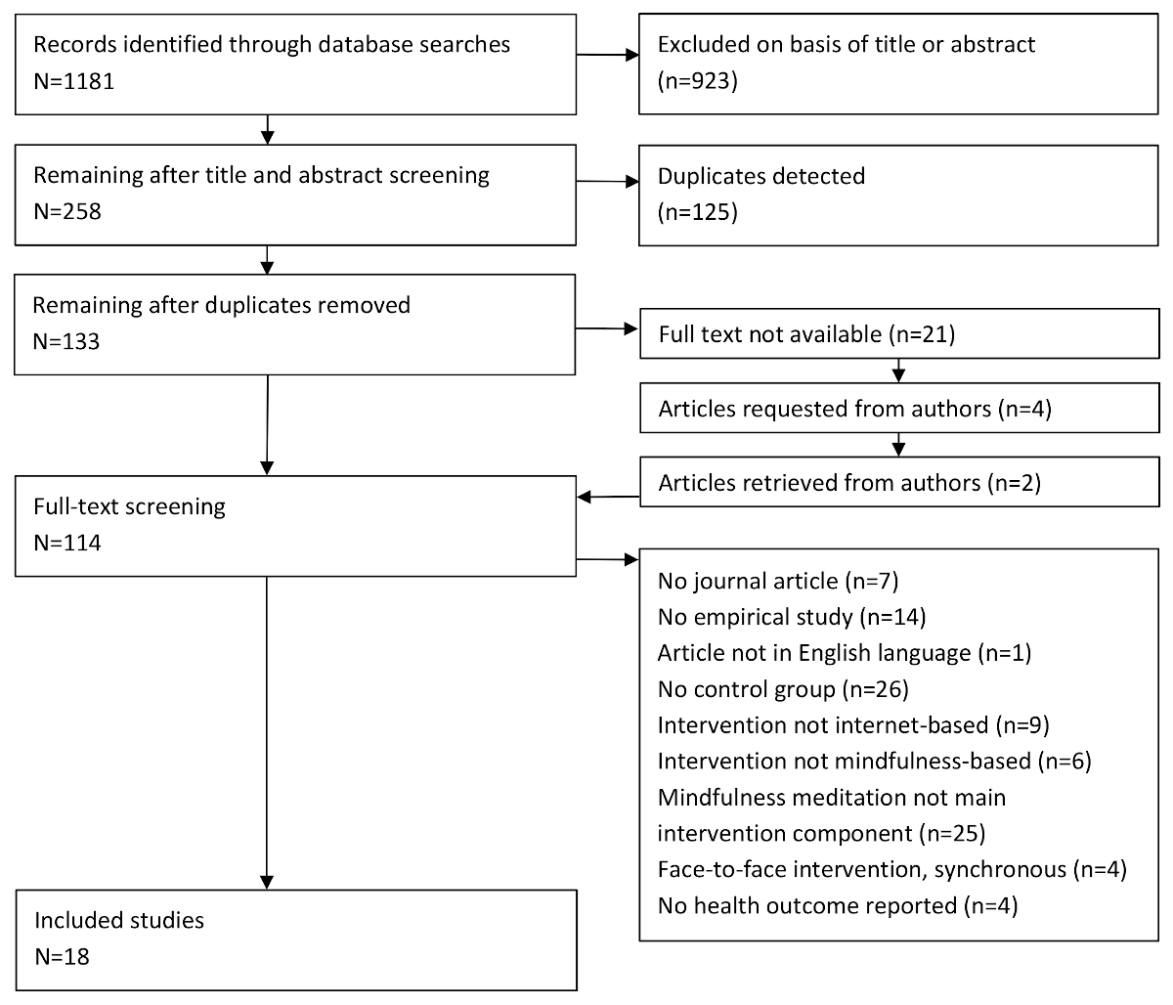
The study selection process in phase 1.

The search process from the keyword search to the final study selection is visualized in Figure 2. The systematic literature search across the 4 databases revealed 1181 results. In the title and abstract screening, 258 search results were identified as relevant, of which 125 were identified as duplicates. Of the remaining 133 studies, 112 were accessible. Of the nonaccessible 21 studies, 7 were identified as dissertations and conference papers, 3 did not employ control groups, and 2 were nonempirical. Of the remaining 5 nonaccessible studies, 1 study did not include author information. The remaining 4 nonaccessible studies were requested directly from the authors via email, with a return of 2 studies that were then added to the pool of studies for a full-text screening. This procedure resulted in 114 studies that were subjected to a full-text screening with regard to the defined inclusion and exclusion criteria. Of these 114 studies, 18 studies matched the inclusion criteria and were, therefore, included in this systematic review [29-46].

#### General Description and Intervention Effectiveness

The general descriptions of the characteristics of the 18 studies included in phase 1 are reported in detail for each study (authors, year of publication, study design, follow-up measures, sample size, age, and indication) in Multimedia Appendix 1. The quality scores in terms of risk bias are also reported in Multimedia Appendix 1. In summary, 3 studies achieved a quality score of 14, 9 a score of 13, 3 a score of 12, 1 a score of 10, and 2 a score of 9. The majority of interventions (n=12) were aimed at psychological symptoms such as stress, anxiety, depression, and general well-being. A total of 4 interventions were aimed at physiological symptoms (eg, fibromyalgia, chronic pain, and heart disease). A total of 2 interventions were aimed at work-related issues (eg, work-life balance and work-related well-being).

A detailed overview of intervention effectiveness (ie, outcome measures, control groups, within- and between-group effects, and computed effectiveness ratings or ERs) is reported in Multimedia Appendix 2. Upon applying the criteria for defining intervention effectiveness [21], 11 studies were classified as more effective, 6 as less effective, and 1 as ineffective.

#### Intervention Design and Effectiveness

The intervention design, duration and scheduling, and adherence and acceptance of the interventions are reported in detail in Multimedia Appendix 3. For an overview of intervention design relative to its effectiveness, ERs are reported again in this overview.

#### Intervention Ratings for 4-Component Instructional Design Components

Tables 3 and 4 report the ERs for the interventions identified in phase 1 relative to the ratings for their design components in the 4C/ID model, namely, LT, SI, PTP, and JIT. For an overview of the exact scoring rules, refer to Table 2 in the Methods section. All studies were rated by 2 independent raters. Initial interrater reliability was determined with the intraclass correlation coefficient (ICC). The ICC was 0.969 with a 95% confidence interval 0.950 to 0.981 (F72,2=33.077; *P*<.01). A total of 4 cases in which raters 1 and 2 differed were identified and discussed until consensus was reached.

**Table 3:** Intervention effectiveness and ratings for 4-component instructional design components in phase 1.

Author (year), country	Effectiveness rating	Learning task	Supportive information	Part-task practice	Just-in-time information	Duration (weeks)
Allexandre et al (2016), United States [29]	++^a^	2	2	0	2	8
Boettcher et al (2014), Sweden [30]	++	2	2	2	1	8
Carissoli et al (2015), Italy [31]	+^b^	2	0	0	0	3
Cavanagh et al (2013), United Kingdom [32]	++	2	2	0	2	2
Davis and Zautra (2013), United States [33]	++	2	2	2	0	6
Dimidjian et al (2014), United States [34]	++	1	2	1	0	8
Dowd et al (2015), Ireland [35]	++	2	2	0	2	6
Glück and Maercker (2011), Austria [36]	+	2	0	0	1	2
Gotink et al (2017), The Netherlands [37]	++	2	1	2	2	17
Howells et al (2014), United Kingdom [38]	+	2	1	0	0	1.5
Ly et al (2014), Sweden [39]	+	2	2	0	2	8
Mak et al (2015), China [40]	+	2	2	2	1	8
Michel et al (2014), Germany [41]	++	2	2	0	1	3
Morledge et al (2013), United States [42]	++	2	2	2	2	8
Noguchi et al (2017), Japan [43]	+	1	1	0	1	5
O’Leary and Dockray (2015), Ireland [44]	0^c^	1	0	1	0	3
Querstret et al (2017), United States [45]	++	2	2	2	1	4
Younge et al (2015), The Netherlands [46]	++	2	2	2	2	12

^a^++ indicates that the intervention was rated as more effective.

^b^+ indicates that the intervention was rated as less effective.

^c^0 indicates that the intervention was rated as ineffective.

**Table 4:** Average ratings for 4-component instructional design components by effectiveness in phase 1.

Intervention code	Average score				
	Learning task	Supportive information	Part-task practice	Just-in-time information	Duration (weeks)
More effective (n=11)	1.91	1.91	1.18	1.35	7.45
Less effective (n=6)	1.83	1.00	0.33	0.83	4.58
Ineffective (n=1)	1.00	1.00	0.00	1.00	3.00

On the basis of the average ratings reported above, we drew the following conclusions that served as the hypothesis for phase 2 of CIS:

- More effective interventions implement formal mindfulness exercises of varying content at least twice per week. Moreover, they continuously provide supportive educational material or reflection exercises, or both. Furthermore, they provide informal PTP opportunities about once per week, provide JIT in the form of reminders at least once per week or when adherence declines, and last for an average of 7 weeks.
- Less effective interventions also implement formal mindfulness exercises of varying content at least twice per week, but SI is only provided once or upon demand. The interventions contain hardly any informal PTP and provide JIT in the form of reminders only about once per week or when adherence declines. The average duration of less effective interventions is 5 weeks.
- Ineffective interventions implement formal mindfulness exercises less than twice per week and provide no SI, no JIT, and hardly any PTP opportunities. The average duration of ineffective interventions is 3 weeks.

### Phase 2: Representative Sampling

The search and review strategy for phase 2 is described in detail in the Methods section. The search in phase 2 yielded 14 additional empirical studies that matched our search criteria [47-60]. The 14 studies and their interventions are described in detail further.

#### General Description and Intervention Effectiveness

The general descriptions of the characteristics of the 14 studies (eg, authors, year of publication, study design, follow-up measures, sample size, age, and indication) included in phase 2 are reported in detail for each study in Multimedia Appendix 4. The quality scores in terms of risk bias are also reported in Multimedia Appendix 4. In summary, 2 studies achieved a quality score of 14, 1 a score of 13, 3 a score of 12, 2 a score of 10, 2 a score of 9, and 1 a score of 7. The vast majority of interventions (n=12) were again aimed at psychological symptoms, such as stress, anxiety, depression, and general well-being. Only 1 intervention was aimed at weight in relation to stress.

A detailed overview of intervention effectiveness (eg, outcome measures, control groups, within- and between-group effects, and computed ERs) is reported in Multimedia Appendix 5. In summary, 6 of the 14 studies were classified as more effective, 6 as less effective, and 2 as ineffective.

#### Intervention Design and Effectiveness

The intervention design, duration and scheduling, and adherence and acceptance of the interventions are reported in detail in Multimedia Appendix 6. For an overview of intervention design relative to its effectiveness, ERs are reported again.

#### Intervention Ratings for 4-Component Instructional Design Components

Tables 5 and 6 below report the ERs for the interventions identified in phase 2, relative to the ratings for their design components in the 4C/ID model, namely, LT, SI, PTP, and JIT. For an overview of the exact scoring rules, refer to Table 2. All studies were rated by 2 independent raters. Initial interrater reliability was determined with the ICC. The ICC was 0.918 with a 95% confidence interval from 0.854 to 0.953 (F56,2=13.167; *P*<.01). A total of 5 cases in which raters 1 and 2 differed were identified and discussed until consensus was reached.

As the same intervention was implemented across 3 separate studies, an average rating score across those 3 studies was computed for each of the instructional design components. Hence, the number of reported interventions (N=12) does not match the number of reviewed studies (N=14) in Table 6 for phase 2.

**Table 5:** Intervention effectiveness and ratings for 4-component instructional design components in phase 2.

Author (year), country	Effectiveness rating	Learning task	Supportive information	Part-task practice	Just-in-time information	Duration (weeks)
Antonson et al (2018), Sweden [47]	0	2	1	0	0	8
Bostock et al (2018), United Kingdom^a^ [48]	++^b^	2	2	1	2	8
Champion et al (2018), United Kingdom^a^ [49]	++	2	2	1	1	4
Joyce et al (2019), Australia [50]	+^c^	2	2	2	2	6
Kvillemo et al (2016), Sweden [51]	+	1	2	2	1	8
Lindsay et al (2018), United States [52]	+	2	1	2	2	2
Lyzwinski et al (2019), Australia [53]	++	2	2	2	2	11
Ma et al (2018), China [54]	+	1	2	1	1	8
Nguyen-Feng et al (2017), United States [55]	+	1	2	0	2	4
Querstret et al (2018), United Kingdom [56]	++	2	2	2	1	4
Shore et al (2018), United Kingdom [57]	++	2	2	2	2	2
van Emmerik et al (2018), The Netherlands [58]	++	2	2	1	2	8
Wahbeh and Oken (2016), United States [59]	0^d^	2	2	2	0	6
Yang et al (2019), United States^a^ [60]	++	2	1	1	1	4

^a^Intervention Headspace.

^b^++ indicates that the intervention was rated as more effective.

^c^+ indicates that the intervention was rated as less effective.

^d^0 indicates that the intervention was rated as ineffective.

**Table 6:** Average ratings for 4-component instructional design components by effectiveness in phase 2.

Intervention code	Average Rating Score for each of the 4 instructional design components				
	Learning task	Supportive information	Part-task practice	Just-in-time information	Duration (weeks)
More effective (n=5)	2.00	1.93	1.60	1.67	5.86
Less effective (n=5)	1.40	1.80	1.40	1.60	5.6
Ineffective (n=2)	2.00	1.50	1.00	0.00	7.00

On the basis of the average ratings reported above, we drew the following conclusions for phase 2 of CIS:

- More effective interventions implement formal mindfulness exercises of varying content at least twice per week. Moreover, they continuously provide supportive educational material or reflection exercises, or both. Furthermore, they provide PTP opportunities implemented at least twice per week, and provide JIT in the form of reminders for each practice, which are sometimes adjustable or contain prompts for self-monitoring. The average duration of more effective interventions in phase 2 is 6 weeks.
- Less effective interventions implement formal mindfulness exercises less than twice per week. Moreover, they continuously provide supportive educational material or reflection exercises, or both. Furthermore, they provide PTP opportunities about once per week, and provide JIT in the form of reminders for each practice, which are sometimes adjustable or contain prompts for self-monitoring. The average duration of less effective interventions is 6 weeks.
- Ineffective interventions implement formal mindfulness exercises of varying content at least twice per week. Moreover, they continuously provide supportive educational material or reflection exercises, or both. Furthermore, they provide PTP opportunities about once per week, but they provide no JIT. The average duration of ineffective interventions is 7 weeks.

## Discussion

### Principal Findings

This paper addressed the following 2 research questions:

1. *Which instructional design components can be identified in existing internet-based mindfulness interventions?*
2. *How can these design components be classified relative to the intervention effectiveness?*

With reference to the 4C/ID model [20], the CIS method [26] was applied across 2 phases (diversity sampling and representative sampling) to source for relevant literature. We determined the effectiveness of the identified studies in accordance with the criteria for defining intervention effectiveness (Table 1) [21] and rated the effectiveness of the interventions relative to intervention design components in accordance with the system we developed for this paper (Table 2). Phase 1 yielded 18 studies with 18 different interventions (Multimedia Appendices 1-3); phase 2 yielded an additional 14 studies with 12 different interventions (Multimedia Appendices 4-6). In the 32 studies identified across phases 1 and 2, 5 achieved a risk bias quality score of 14, 10 a score of 13, 6 a score of 12, 3 a score of 10, 4 a score of 9, and 1 a score of 7. The majority of studies (n=24) aimed at psychological symptoms, such as stress, anxiety, depression, or general well-being. A total of 5 studies aimed at physiological symptoms related to fibromyalgia, chronic pain, heart disease, and body weight. A total of 2 interventions aimed at work-life balance and work-related well-being. The 32 studies contained 30 different interventions. Of those interventions, 17 classified as more effective, 12 as less effective, and 3 as ineffective. When comparing the results of phases 1 and 2, the following picture emerges.

More effective interventions consistently implemented formal mindfulness exercises of varying content at least twice per week, continuously provided educational and supportive material and/or reflection exercises, provided informal PTP opportunities at least once per week, and provided JIT in the form of reminders at least once per week or when adherence declined. The average duration of more successful interventions across phases 1 and 2 was 6.5 weeks.

Less effective interventions also consistently implemented formal mindfulness exercises of varying content at least twice per week. These provided SI at least once and contained informal PTP opportunities about once per week or less. JIT in the form of reminders was provided at least once per week or when adherence declined. The average duration of less effective interventions across phases 1 and 2 was 5.5 weeks.

Ineffective interventions implemented formal mindfulness exercises at least once per week but varied strongly in the level of SI they provided. These provided informal PTP opportunities only up to once per week and did not provide any JIT (ie, no reminders). The average duration of ineffective interventions across phases 1 and 2 was 5 weeks.

In summary, the overwhelming majority of internet-based mindfulness interventions are more or less effective, and the effectiveness of the interventions increases with the level of support provided by instructional design components. The difference between effective and ineffective interventions is the presence of JIT in the form of reminders, in addition to the availability of LTs (ie, formal mindfulness exercises), SI (ie, educational material and/or reflection exercises), and PTP (ie, reminders to practice and/or prompts for self-monitoring). We thus conclude that to be effective at all, internet-based mindfulness interventions must contain all 4 4C/ID design components. The difference between more effective and less effective interventions is the presence of continuous support with information in the form of educational materials and/or self-reflection exercises, as compared with SI that is optional or only provided once. The duration of the interventions alone does not seem to be systematically related to intervention effectiveness when taking into account the findings of phases 1 and 2 of our CIS. Phase 1 suggested that ineffective interventions are shorter (average duration 3 weeks) than less and more effective interventions (average durations between 5 and 7 weeks). However, this notion was not supported in phase 2 that revealed no systematic differences in intervention effectiveness based on duration.

### Limitations

This CIS is limited, naturally, by its scope and search criteria. For example, only studies in international peer-reviewed journals were considered, and there might be a number of interesting dissertations, conference presentations, and thesis projects, which may contribute to a further understanding of the design components and effectiveness of internet-based mindfulness interventions, that remained unidentified in this review. In addition, there are limitations regarding search terms and sensitivity. As this literature review relied heavily upon relatively broad search criteria such as *meditation*, it attempted to detect studies on internet-based mindfulness interventions with high sensitivity. However, it is likely that a number of studies remained undetected by the applied search strategy. We addressed this issue by extending our original searches with an additional hand search in phase 2, thus identifying an additional 14 studies. In contrast to systematic reviews, the CIS method [26] does not require an exhaustive literature search. Nonetheless, we made efforts to identify all published papers that matched the focus of this investigation.

On the level of the individual studies, limitations relate to small sample sizes in some cases and uneven gender distributions. Regarding statistical analyses, some studies reported only results for per-protocol analyses and not for intention-to-treat analyses that attempt to reduce bias resulting from missing data of dropouts and withdrawals. However, high attrition rates and intention-to-treat analyses may diminish the power to detect group differences [61]. Risks of bias can also be derived from the requirement for participants to have regular access to an internet-enabled device, which points to the *digital divide*, that is, the gap in internet access between the general population and underserved populations [18] that might be in more need of health-improving interventions [18]. Another potential source for bias concerns the publication bias, that is, the circumstance that predominantly studies with significant effects are published, and studies with nonsignificant results for the same interventions may go unnoticed [21].

In terms of interpreting the results of this CIS, we noticed that it would be insightful to contrast the instructional design components of experimental and active control groups. Although addressing this interesting question exceeds the scope of this investigation, it provides ample incentive and opportunity for future research.

### Comparison With Previous Studies

In the past 4 years, 7 major reviews were published on the topic of internet-based mindfulness interventions [9-15]. Those reviews focused on intervention effects and revealed heterogeneous, but predominantly encouraging, results in support of internet-based mindfulness interventions for a variety of mental and physical health conditions [9-15]. Of these 7 reviews, 4 [10,12,14,15] specifically point to design components as the potential sources of variance in intervention effectiveness and call for research to address this issue. This paper serves the purpose.

In line with the existing reviews [9-15], we also found that the majority of the interventions were more or less effective, particularly with regard to mental health. This investigation extends previous studies by providing insight into the instructional design of the implemented interventions relative to the intervention effectiveness. In addition, the rating system for the instructional design components, which we developed based on the 4C/ID model for the purposes of this investigation, is now available to other researchers as a useful tool to classify design components of interventions.

### Conclusions

The vast majority of internet-based mindfulness interventions that were identified for this CIS were more or less effective in producing significant changes in the assessed outcome measures. The main difference between effective and ineffective interventions is the presence of JIT in the form of reminders, in addition to the availability of the other 3 design components—LTs, SI, and PTP. The main difference between more effective and less effective interventions is the presence of continuous support with information in the form of educational materials and/or self-reflection exercises, as compared with SI that is optional or only provided once. In summary, we conclude that the effectiveness of the interventions increases with the level of support provided by the instructional design components.

## Appendix

Multimedia Appendix 1 Characteristics of the included studies in phase 1.

Multimedia Appendix 2 Intervention effectiveness of the included studies in phase 1.

Multimedia Appendix 3 Intervention design of the included studies in phase 1.

Multimedia Appendix 4 Characteristics of the included studies in phase 2.

Multimedia Appendix 5 Intervention effectiveness of the included studies in phase 2.

Multimedia Appendix 6 Intervention design of the included studies in phase 2.

## Abbreviations

4C/ID: 4-component instructional design
CIS: critical interpretive synthesis
ER: effectiveness rating
ICC: intraclass correlation coefficient
JIT: just-in-time information
LT: learning task
PTP: part-task practice
SI: supportive information
