# Supplementary material for: Relating Instructional Design Components to the Effectiveness of Internet-Based Mindfulness Interventions: A Critical Interpretive Synthesis
Source: J Med Internet Res. 2019 Nov 27;21(11):e12497. doi: 10.2196/12497 (PMC6906627; doi:10.2196/12497)
Supplement: Multimedia Appendix 1 [file jmir_v21i11e12497_app1.pdf]

Multimedia Appendix 1

Characteristics of the included studies in phase 1

Author (Year), Country	Design	Quality score	Follow-Up	N (% female)	M Age (SD)	Specifics	Indication
Allexandre et al. (2016), USA	RCT	13	16 weeks, 1 year	161 (83.2)	40.0 (12.6)	Community sample	Stress
Boettcher et al. (2014), Sweden	RCT	13	6 months	91 (71.4)	38.0 (10.3)	Clinical sample	Anxiety
Carissoli et al. (2015), Italy	RCT	9	-	56 (57.0)	38.1 (6.9)	Community sample	Well-being
Cavanagh et al. (2013), UK	RCT	12	-	104 (88.4)	24.7 (6.4)	Community sample	Well-being
Davis & Zautra (2013), USA	RCT	12	-	79 (98.0)	46.1 (n.a.)	Clinical sample	Fibromyalgia
Dimidjian et al. (2014), USA	QT	13	2.5 months, 6 months	200 (71.5)	IG: 47.7, CG: 51.0	Clinical sample	Depression
Dowd et al. (2015), Ireland	Pilot RCT	13	6 months	124 (90.3)	44.5 (12.3)	Clinical sample	Chronic pain
Glück & Maercker (2011), Austria	Pilot RCT	13	3 months	49 (73.5)	IG: 33.7 (12.7), CG: 37.2 (14.4)	Community sample	Psychological distress
Gotink et al. (2017), Netherlands	RCT	14	-	324 (37.3)	43.2 (13.9)	Clinical sample	Heart disease
Howells et al. (2014), UK	RCT	10	-	194 (86.6)	40.7 (10.6)	Community sample	Well-being
Ly et al. (2014), Sweden	RCT	12	6 months	81 (70.3)	36.0 (10.8)	Clinical sample	Depression
Mak et al. (2015), China	RCT	13	3 months	321 (66.3)	22.8 (6.5)	Community sample	Well-being
Michel et al. (2014), Germany	RCT	13	5 weeks	246 (71.1)	41.4 (9.4)	Community sample	Work-life balance
Morledge et al. (2013), USA	RCT	13	12 weeks	551 (88.9)	n.a.	Mixed clinical and community sample	Stress
Noguchi et al. (2017), Japan	RCT	14	12 weeks, 18 weeks	974 (50.1)	43.7 (11.3)	Clinical sample	Depression
O'Leary & Dockray (2015), Ireland	RCT	9	5 weeks	62 (100)	28.35 (6.7)	Not specified	Well-being
Querstret et al. (2017), USA	RCT	14	3 months, 6 months	118 (80.5)	40.7 (10.5)	Community sample	Work-related well-being

Younge et al. (2015), Netherlands	RCT	13	-	324 (46.3)	43.2 (14.1)	Clinical sample	Heart disease

QT … quasiexperimental open trial
RCT … randomized controlled trial
