# Supplementary material for: Relating Instructional Design Components to the Effectiveness of Internet-Based Mindfulness Interventions: A Critical Interpretive Synthesis
Source: J Med Internet Res. 2019 Nov 27;21(11):e12497. doi: 10.2196/12497 (PMC6906627; doi:10.2196/12497)
Supplement: Multimedia Appendix 2 [file jmir_v21i11e12497_app2.pdf]

Multimedia Appendix 2

Intervention effectiveness of the included studies in phase 1

Author (Year), Country	Outcome measures	Control groups	Within effects	Between effects	ER
Allexandre et al. (2016), USA	Perceived stress, emotional exhaustion, personal efficacy, mindfulness, emotional well-being, emotional role-functioning, vitality	2 x ACG, 1 x WLC	decrease in perceived stress, emotional exhaustion in post and first follow-up (d 0.3 - 1.1); increase in mindfulness, emotional variables and vitality in post and first follow-up (d 0.3 – 1.1); second follow-up significant increase in vitality and emotional role-functioning (d 0.5 - 1.1)	superior to WLC in perceived stress, emotional well-being and vitality (d 0.6 – 0.7); not more effective than ACGs and less effective than ACGs in perceived stress and emotional variables (d 0.4 - 0.6)	++
Boettcher et al. (2014), Sweden	Anxiety, Depression, Quality of life, Insomnia	1 x ACG	decrease in anxiety, depression, insomnia in post and first follow-up (d 0.82 – 1.58); increase in quality of life in post and first follow-up (d 0.53 - 0.64)	superior to ACG in anxiety, depression, quality of life, insomnia (d 0.36 - 0.99)	++
Carissoli et al. (2015), Italy	Perceived stress, Heart rate	1 x ACG 1 x WLC	Significant decrease in perceived stress and heart rate in post	No significant between effects	+
Cavanagh et al. (2013), UK	Mindfulness, Perceived stress, depression + anxiety	1 x WLC	decrease in perceived stress and depression + anxiety in post (d 0.24 - 0.37); increase in mindfulness in post (d. 0.27)	Superior to WLC in mindfulness, perceived stress, depression + anxiety (d 0.4 - 0.62)	++
Davis & Zautra (2013), USA	Pain, pain coping efficacy, positive and negative affect, loneliness, social activity engagement, family stress, family enjoyment, stress coping efficacy	1 x ACG	Decrease in negative affect, loneliness, family stress in post; increase in pain coping efficacy, stress coping efficacy in post	Superior to ACG in pain coping efficacy, positive affect, loneliness, social activity engagement, family enjoyment, stress coping efficacy (eta^2 0.009 - 0.060)	++
Dimidjian et al. (2014), USA	Depression, rumination, mindfulness	1 x ACG	Decrease in depression (d. 0.78), decrease in subgroup analysis with residual depressive symptoms (d. 1.54), significant decrease in rumination in post; increase in mindfulness in post	Superior to ACG in depression and subgroup analysis with residual depressive symptoms (d 0.78 - 1.79)	++

Dowd et al. (2015), Ireland	Pain interference, pain intensity, pain catastrophizing, pain acceptance, mindfulness, life satisfaction, patient impression of change	1 x ACG	Significant decrease in pain interference, pain intensity (pain "right now"), pain catastrophizing, mindfulness (d 0.42 - 0.76); increase in pain acceptance, life satisfaction (d 0.58 - 0.90)	Superior to ACG in life satisfaction, patient impression of change (d 0.41 - 0.59)	++
Glück & Maercker (2011), Austria	General distress, perceived stress, mindfulness, emotion regulation + mood	1 x WLC	Significant increase in emotion regulation + mood in post and first follow- up (d 0.43)	interaction effect for participants reporting higher vs. lower distress before the program (d = 0.85)	+
Gotink et al. (2017), Netherlands	Exercise capacity, Blood pressure, Mental + physical functioning, anxiety and depression	1 x WLC	improved exercise capacity, blood pressure, mental functioning and depression	Superior to WLC in exercise capacity, blood pressure, mental + physical functioning, anxiety and depression (d 0.18 - 0.22)	++
Howells et al. (2014), UK	Life satisfaction, Flourishing scale, positive and negative affect, depression	1 x ACG	Significant decrease in depression in post; significant increase in positive affect in post (eta^2 0.071)	No between effects	+
Ly et al. (2014), Sweden	Depression (BDI-II), Depression (PHQ-9), Anxiety, Quality of life, Acceptance and action, Recovery rate	1 x ACG	Decrease in Depression (BDI –II), Depression (PHQ – 9), Anxiety in post and first follow up (d 0.5 - 1.21), Decrease in quality of life, acceptance and action in first follow up (d 0.75 - 0.87); increase in quality of life, acceptance and action in post (d 0.56 - 0.62)	No significant group differences	+
Mak et al. (2015), China	Mindfulness, global mental well-being, life satisfaction, perceived stress, depression + anxiety + stress	1 x ACG<br>1 x WLC	Increase in global mental well-being in post (eta^2 0.09)	No significant between effects except for mindfulness in first follow-up (p 0.05)	+

Michel et al. (2014), Germany	Mindfulness, psychological detachment from work, strain-based family conflict, satisfaction with work-life balance	1 x WLC	Improvement in Mindfulness, psychological detachment from work, strain-based family conflict, satisfaction with work-life balance in post	Superior to WLC in mindfulness, psychological detachment from work, strain-based family conflict, satisfaction with work-life balance (eta^2 0.030 – 0.092)	++
Morledge et al. (2013), USA	Perceived stress, mindfulness, self-acceptance (ASTI), self-acceptance (PWB-SA), vitality, general physical health, general mental health	1 x ACG<br>1 x WLC	Improvement in Perceived stress, mindfulness, self-acceptance (ASTI), self-acceptance (PWB -SA), vitality, general mental health	Superior to WLC in Perceived stress, mindfulness, self-acceptance (ASTI), self-acceptance (PWB-SA), general mental health in post; superior to WLC in vitality in first follow-up; not more effective than ACG	++
Noguchi et al. (2017), Japan	Depression (CES-D), Depression (PHQ-9), generalizes anxiety	1 x ACG<br>1 x WLC	Improvement in both depression indices and anxiety	Superior to ACG and WLC in depression (PHQ-9) in post; less effective than ACG and WLC in depression (CES-D), generalized anxiety in second follow-up	+
O´Leary & Dockray (2015), Ireland	Perceived stress, depression, happiness	1 x ACG<br>1 x WLC	No significant effects	No significant effects	o
Querstret et al. (2017), USA	Work-related rumination, fatigue, sleep quality, mindfulness	1 x WLC	Improvement in rumination, fatigue, sleep, sleep quality	Superior to WLC in sleep quality, work-related rumination, fatigue in post (eta^2 > 0.14)	++
Younge et al. (2015), Netherlands	Exercise capacity and other physiological (weight, heart-rate, blood pressure, respiratory rate, blood) and psychological (general health, quality of life, perceived stress, depression and anxiety, social support) measures	ACG	Decrease in heart rate, systolic blood pressure, perceived stress and depression, increased exercise capacity	Superior to ACG in exercise capacity, heart rate, blood pressure, depression and perceived stress (d 0.17 - 0.21)	++

ACG … active comparison group
ASTI … Adult Self-Transcendence Inventory
BDI II … Beck’s Depression Inventory II
CES-D … Center for Epidemiologic Studies Depression Scale

CG … control group
ER … effectiveness rating
PHQ-9 … Patient Health Questionnaire for Depression and Anxiety
PWB-SA … Psychological Well-Being-Self Acceptance
WLC … waitlist control group
