# Supplementary material for: Relating Instructional Design Components to the Effectiveness of Internet-Based Mindfulness Interventions: A Critical Interpretive Synthesis
Source: J Med Internet Res. 2019 Nov 27;21(11):e12497. doi: 10.2196/12497 (PMC6906627; doi:10.2196/12497)
Supplement: Multimedia Appendix 3 [file jmir_v21i11e12497_app3.pdf]

Multimedia Appendix 3

Intervention design of the included studies in phase 1

Author (Year), Country	ER	Intervention design	Duration and scheduling	Adherence and acceptance
Allexandre et al. (2016), USA	++	Website “stress free now”	8 weeks	42-52% of participants never logged on; regular access 19% of participants
		LT: Formal mindfulness meditation exercise in audio format, played from website or downloadable in mp3 format		
		SI: Introductory talk in written and audio format on the theme or meditation technique of the week (psycho-education) + daily articles providing an overview of the science underlying the benefits of mindfulness meditation		
		PTP: -		
		JIT: Email reminders twice weekly to access website and practice mindfulness		
Boettcher et al. (2014), Sweden	++	Website	8 weeks, 6 days of the week	average task completion 44 out of 96 exercises; intervention group more satisfied than control
		LT: two daily formal mindfulness meditation exercises, at 10 minutes each		
		SI: weekly 20-minute introduction video (psycho-education), short reflection exercises		
		PTP: instructions on how to apply mindfulness in daily life		
		JIT: standardized email reminder at week 4		
Carissoli et al. (2015), Italy	+	Smartphone application “It’s Time to Relax”	3 weeks, 2 formal exercises per day	45% of participants practiced at least once per day; the app was rated simple (M = 2.43) and useful (M = 3.65)
		LT: 15 min guided meditations (first week), free mediations (second + third week), at 15 minutes each		
		SI: -		
		PTP: -		
		JIT: -		

Cavanagh et al. (2013), UK	++	Website ‘Learning Mindfulness Online’	2 weeks, daily practice	61% of participants reported to have practiced more than once a week, 26% more than once a day; 87% found program beneficial
		LT: choice of guided mindfulness meditations (10 minutes) for daily practice, with instructions		
		SI: text and video about benefits of mindfulness, information regarding what to expect during practice, help and assistance sections (psycho-education) + reflection exercises		
		PTP: -		
		JIT: Standardized email reminders at 3-day intervals		
Davis & Zautra (2013), USA	++	Website ‘Mindful socioemotional regulation intervention (MSER)’	6 weeks, 1 formal exercise per day	average access of 8.23 modules,
		LT: formal mindfulness exercises, 12 consecutive modules at 15 minutes each		
		SI: psycho-educative content in written and audio formats centering on particular mindfulness topic (e.g. acceptance of emotions, mindful living with pain) + reflection exercises		
		PTP: daily informal mindfulness practice		
		JIT: -		
Dimidjian et al. (2014), USA	++	Website ‘Mindful Mood Balance (MBB)’	8 weeks, 1 session per week	42% of participants reported completing all 8 sessions, 53% completed at least 4; mean weekly frequency of formal practice was 2.56 times, mean weekly frequency of brief daily practice practice was 8.91 times
		LT: formal mindfulness practice		
		SI: audio guides, informational material (psycho-education) + reflection exercises		
		PTP: video-based vicarious learning (e.g. video interaction between instructors and participants in a mindfulness class)		
		JIT: -		

Dowd et al. (2015), Ireland	++	Website 'Mindfulness in Action'	6 weeks, 2 sessions per week, daily formal exercises	mean number of sessions viewed 11.2, with 74% of participants reporting viewing all of the sessions; 73.9% reported meditating between 6 and 20 minutes each day
		LT: audio-recorded daily meditation exercises		
		SI: prerecorded audiovisual presentation to build mindfulness skills, information on how to cultivate and sustain positive emotional experiences, particularly within social relationships (psycho-education) + reflection exercises		
		PTP: -		
		JIT: standardized email reminders twice weekly		
Glück & Maercker (2011), Austria	+	Website	2 weeks, 6 days per week	64.3% participated for 6 or more days; at post, 73.5% and at follow-up, 66.6% of participants stated that they found the program beneficial. 77.2% would recommend the program
		LT: 20 min audio files with guided meditation exercises such as awareness of body sensations, attention to breath, acceptance of upcoming emotions (module 1); flash-animated thought distancing exercise (module 2)		
		SI: -		
		PTP: -		
		JIT: Standardized email reminder after fist module		
Gotink et al. (2017), Netherlands	++	Website (structured mindfulness program)	17 weeks, bi-weekly exercises	Adherence was monitored by whether the questions of the online program were completed
		LT: 10-15 minutes formal mindfulness exercises		
		SI: book on mindfulness (psycho-education)		
		PTP: Informal mindfulness practice in daily life with emphasis on breathing		
		JIT: Bi-weekly reminders via email and standardized text message		

Howells et al. (2014), UK	+	Smartphone application ‘Headspace’	10 days , daily exercise	ratings of task enjoyment were positively correlated with positive affect increase (r=.285)
		LT: 10-minute audio mindfulness exercises<br>SI: 2-minute introduction video (psycho-education)<br>PTP: -<br>JIT: -		
Ly et al. (2014), Sweden	+	Smartphone application	8 weeks	adherence to all 8 weeks was 78% in the mindfulness-based program group,<br>compared to 63% in the control group receiving the behavioral activation program; overall average adherence was 6 weeks
		LT: Audio files with mindfulness exercises of guided and unguided and short (3minutes) or long (30 minutes) formats<br>SI: Short web-based psycho-education + weekly reflection exercises<br>PTP: -<br>JIT: standardized therapist messages every other day + weekly educational emails		
Mak et al. (2015), China	+	Website ‘Mindful living’	8 weeks, formal mindfulness exercises 6 days of the week	average number of days of use 4.34
		LT: Downloadable meditation audios and videos for mindfulness exercises lasting 20-30 minutes each<br>SI: 3-hour workshop to provide overview of mindfulness and website, 30-minute mindfulness lessons at one per week (psycho-education)<br>PTP: daily informal mindfulness practice<br>JIT: Weekly standardized email reminders		

Michel et al. (2014), Germany	++	Website	3 weeks, daily practice	n.a.
		LT: Formal mindfulness exercises of 3-5 minutes		
		SI: 20 minutes weekly psycho-educative information in written format (on weekend)		
		PTP: -		
		JIT: 3 standardized text message reminders or 1 email reminder at beginning of week		
Morledge et al. (2013), USA	++	Website	8 weeks, 1 session per week + formal exercises 5 times per week	for the 115 participants (<35%) completing ≥ 4 weekly logs, meditation practice averaged 4.07 times per week; 45% found the overall program to be very or extremely helpful, 35% found it somewhat helpful, and 19% found it to be little or not at all helpful; mindfulness techniques rated as the most helpful and beneficial component (53%), followed by the daily articles (48%) and the weekly audio lesson (43%)
		LT: Mindfulness meditation exercises in audio format, played from website or downloaded in mp3-format, lasting 20-25 minutes		
		SI: Introductory talk in written and audio format on theme of meditation technique of the week, daily motivational quotes and tips on how to manage stress (psycho-education)		
		PTP: informal mindfulness practice; daily articles on specific meditation technique components		
		JIT: twice-weekly standardized email reminders introducing the theme and meditation of the week		

Noguchi et al. (2017), Japan	+	Website<br><br>LT: 5-minute formal mindfulness exercise where participants conjure up negative<br>feelings based on recent experiences and practice the acceptance of those feelings<br><br>SI: FAQ resource (psycho-education)<br><br>PTP: -<br><br>JIT: email reminders with exercise prompts and instructions	5 weeks, self-paced	n.a.
O´Leary & Dockray (2015), Ireland	o	Website<br><br>LT: 10-15 min formal mindfulness meditation exercise (body-scan)<br><br>SI: -<br><br>PTP: diary for listing thoughts, feelings, and emotions in the present moment<br><br>JIT: n.a	3 weeks	n.a.
Querstret et al. (2017), USA	++	Website<br><br>LT: 10 interactive guided meditation sessions (e.g. body scan, mindful movement) via video<br><br>SI: 3 min videos psycho-education on mindfulness in beginning of each session<br><br>PTP: informal mindfulness techniques (e.g. mindful eating, mindful walking)<br><br>JIT: reminder if course was not accessed for more than a week	4 weeks,<br>1 session per week and daily practice encouraged	average time to program completion 6 weeks, 5 days; all participants completed program within 12 weeks

Younge et al. (2015), Netherlands	++	Website ‘Mindfulness for a Healthier Heart’	12 weeks, 1 module per week; scheduling of formal and informal practice varied from none to daily across modules	115 participants completed at least 50% of the assignment
		LT: formal mindfulness meditation exercises		
		SI: short videos and written text on mindfulness (psycho-education) + self-reflection exercises		
		PTP: practical assignments for mindfulness in daily life		
		JIT: bi-weekly email reminders and standardized text messages		

ER … effectiveness rating
JIT … just-in-time information
LT … learning task
PTP … part-task-practice
SI … supportive information
