# Supplementary material for: Relating Instructional Design Components to the Effectiveness of Internet-Based Mindfulness Interventions: A Critical Interpretive Synthesis
Source: J Med Internet Res. 2019 Nov 27;21(11):e12497. doi: 10.2196/12497 (PMC6906627; doi:10.2196/12497)
Supplement: Multimedia Appendix 4 [file jmir_v21i11e12497_app4.pdf]

Multimedia Appendix 4

Characteristics of the included studies in phase 2

Author (Year), Country	Design	Quality score	Follow-Up	N (% female)	M Age (SD)	Specifics	Indication
Antonson et al. (2018), Sweden	RCT	9	-	202 (70.3)	16.9 (n.a.)	Secondary school students	Well-being
Bostock et al. (2018), UK	RCT	10	16 weeks	238 (59.2)	35.5 (7.7)	Stressed employees	Stress
Champion et al. (2018), UK	Pilot RCT	12	30 days	74 (55.4)	39.1 (5.7)	Community sample	Well-being
Joyce et al. (2019), Australia	Cluster RCT	14	6 months	143 (4.2)	42.3 (8.6)	Healthy fire-fighters	Resilience
Kvillemo et al. (2016), Sweden	Pilot RCT	11	-	90 (?)	n.a.	University students	Well-being
Lindsay et al. (2018), USA	RCT	10	-	153 (67.3)	32.0 (14.0)	Stressed adults	Well-being
Lyzwinski et al. (2019), Australia	RCT	11	-	90 (67.0)	20.2 (n.a.)	University students	Weight and stress
Ma et al. (2018), China	RCT	9	-	192 (58.0)	27.8 (7.9)	Community sample	Psychological distress
Nguyen-Feng et al. (2017), USA	RCT	12	2-3 weeks, 4-5 weeks	365 (75.0)	n.a.	Psychology students	Mental health, Internet use
Querstret et al. (2018), UK	RCT	13	3 months, 6 months	118 (80.5)	40.7 (10.5)	Community sample	Well-being
Shore et al. (2018), UK	RCT	11	1 week	110 (89.0)	32.2 (13.6)	Community sample	Well-being
van Emmerik et al. (2018), NL	RCT	14	3 months	377 (96.0)	44.7 (9.8)	Community sample	Well-being
Wahbeh & Oken (2016), USA	Pilot RCT	7	-	46 (58.0)	42.0 (14.0)	Community sample	Well-being
Yang et al. (2019), USA	RCT	12	1 month	88 (56.0)	25.1 (n.a.)	Medical students	Stress

RCT … randomized controlled trial
