# Supplementary material for: Relating Instructional Design Components to the Effectiveness of Internet-Based Mindfulness Interventions: A Critical Interpretive Synthesis
Source: J Med Internet Res. 2019 Nov 27;21(11):e12497. doi: 10.2196/12497 (PMC6906627; doi:10.2196/12497)
Supplement: Multimedia Appendix 5 [file jmir_v21i11e12497_app5.pdf]

Multimedia Appendix 5

Intervention effectiveness of the included studies in phase 2

Author (Year), Country	Outcome measures	CG	Within effects	Between effects	ER
Antonson et al. (2018), Sweden	general psychiatric health, sleep quality, perceived stress	ACG, WLC	No significant changes due to low compliance	No significant differences due to low compliance	o
Bostock et al. (2018), UK	psychological well-being, job strain, blood pressure	WLC	increase in well-being, pos. emotions, anxiety, depressive, job strain, social support, marginally significant decrease in blood-pressure ($p < .05$)	improvement in well-being, distress, job-strain (job control), perception of social support (eta^2 .005 - .065)	++
Champion et al. (2018), UK	Life satisfaction, perceived stress, resilience	WLC	improved life satisfaction, stress, resilience at day 10 and day 30 (d = 0.57, 1.42, 0.63); most improvement at 10-day assessment	increased life-satisfaction, less perceived stress, more resilience; more impact after 30 than after 10 days; effect sizes completers only d 0.16 - 0.32 (10 day), d 0.60 - 1.53 (30 day)	++
Joyce et al. (2019), Australia	resilience, acceptance and mindfulness skills, resilience resources (optimism, coping, sense of purpose)	ACG	increase in resilience post and follow-up ($p = .008$)	6 month-follow up superior improvement in resilience ($p = .002$), group differences in resilience resources (use of support, optimism) ($p < .05$), no group differences in other secondary measures	+
Kvillemo et al. (2016), Sweden	Psychological well-being, depression symptoms	ACG	small significant increase in well-being (d = 0.2), no significant change in depressive symptoms	no significant differences	+
Lindsay et al. (2018), USA	positive and negative affect (momentary and end-of-day)	2 x ACG	significant increase in overall positive affect (d = 0.70), subfacets happiness, calm and vigor (d .25 - .65); no significant decrease in negative affect	Superior to both controls in overall positive affect (g = .46, .71), subfacets happiness and calm (g = .54, .45); no significant differences in negative affect	+

Lyzwinski et al. (2019), Australia	weight, stress, mindfulness, mindful eating, physical activity, eating behavior	ACG	decreased stress level ($p = .02$), lower emotional and uncontrolled eating ($p = .02$), higher mindfulness ($p < .001$) and mindful eating ($p < .001$)	superior improvement in mindfulness, stress, mindful eating and eating behavior (eta^2 0.014 – 0.236)	+
Ma et al. (2018), China	psychological distress (depression, anxiety)	2 x ACG, WLC	improvement in mindfulness, emotion regulation, depression (d 0.48-0.91)	less effective than guided mindfulness intervention with group discussion; not significantly more effective than control group	+
Nguyen-Feng et al. (2017), USA	self-report stress, anxiety, depression, perceived stress Indication: lack of mental health resources, high Internet use	2 x ACG	for no-interpersonal-trauma (IPT) participants decrease in stress, depression and anxiety from pre to post, first and second follow-up (d -.10 – -.85); for IPT participants no significant positive effects	no significant group differences for no-IPT participants; for IPT participants mindfulness only intervention least effective (d .12 – .50)	+
Querstret et al. (2018), UK	perceived stress, depression and anxiety	WLC	significant decrease in perceived stress, depression and anxiety in Post-treatment and both follow-ups (eta^2 0.34 – 0.51)	superior to control group in all three outcome variables (d -1.06 – -1.25)	++
Shore et al. (2018), UK	mindfulness and paranoia Indication: reduce levels of paranoia, increase mindfulness	WLC	significant decrease in paranoia (post d = .060, follow-up d = 0.80)	improved reduction in paranoia and mindfulness (post d = .074, follow-up d = 0.62)	++
van Emmerik et al. (2018), Netherlands	mindfulness, quality of life, general psychiatric symptomatology, self-actualization	WLC	in posttest increase in mindfulness (d 0.32 - 0.92), quality of life (d 0.44 – 0.92) and self-actualization (d = 0.39), decrease in psychiatric symptomatology (d = - 0.86); maintained in follow-up (d 0.09 - 0.92)	improvement in mindfulness (d = .077), psychiatric symptoms (d = -0.68), increase in psychological quality of life (d = 0.38); not significant for self-actualization and physical health	++

Wahbeh & Oken (2016), USA	quality of life, self-efficacy, depressive symptoms, sleep, stress, mindfulness, home practice behavior	ACG	no improvement on health outcomes; significant increase in home practice behavior (p .01 - .05)	no differences between groups on health outcomes; significantly more home practice days	o
Yang et al. (2019), USA	perceives stress, mindfulness, general well-being	WLC	increase in three subscales of mindfulness ($p < .05$), , decrease in perceived stress ($p = .04$)	significant increase of well-being in post and follow-up ($p = .04$), perceived stress ($p = .02$)	++

ACG … active comparison group
CG … control group
ER … effectiveness rating
WLC … waitlist control group
