# Supplementary material for: Relating Instructional Design Components to the Effectiveness of Internet-Based Mindfulness Interventions: A Critical Interpretive Synthesis
Source: J Med Internet Res. 2019 Nov 27;21(11):e12497. doi: 10.2196/12497 (PMC6906627; doi:10.2196/12497)
Supplement: Multimedia Appendix 6 [file jmir_v21i11e12497_app6.pdf]

Multimedia Appendix 6

Intervention design of the included studies in phase 2

Author (Year), Country	ER	Intervention design	Duration and scheduling	Adherence and acceptance
Antonson et al. (2018), Sweden	o	Mindfulness based self-help program<br>LT: video or audio material of 10 min mindfulness meditation technique<br>SI: - (only optional email contact to authors)<br>PTP: -<br>JIT: -	8 weeks, twice a day, 6 days per week	15 of 95 logged in to the intervention, only 1 completer
Bostock et al. (2018), UK	++	„Headspace“ smartphone application<br>LT: 10-20 min guided audio meditations (45 sessions)<br>SI: 1 hour in person introductory talk about meditation, introductory videos in app<br>PTP: probably there is some, but it is not described<br>JIT: in-app reminder, weekly study reminder	8 weeks, one meditation per day	on average 16.6 sessions completed, 11 % did not use the app, 75% completed six sessions
Champion et al. (2018), UK	++	„Headspace“ smartphone application<br>LT: 10-20 min guided audio meditations (30 sessions)<br>SI: educational videos and animations in app<br>PTP: probably there is some, but it is not described<br>JIT: probably there is some, but it is not described; encouraging mail after day 10	10 and 30 days, one meditation per day	on average 6.21 interactions with the app in 10 days, 75% of participants rated the intervention as enjoyable or very enjoyable

Joyce et al. (2019), Australia	+	Resilience@Work (RAW) Mindfulness Program, used on tablets at work	3.5 – 6 weeks, 6 sessions with 3 days breaks in between	6-week 55.2%, 6-month 48.3% overall adherence to the study, on average participants completed 3.5 sessions, 37% completed 5-6 sessions
		LT: 8 weekly modules, audio files 15-30 min exercises (formal exercises from MBSR)		
		SI: few pages of text about theoretical foundations of mindfulness in each module; possibility to phone call or e-mail study coordinators		
		PTP: informal practice exercises (e.g. awareness of routine activities)		
		JIT: weekly reminder that new module is available; additional reminders if no log-in for 14 days or weekly assessment not responded		
Kvillemo et al. (2016), Sweden	+	Internet-based mindfulness training program, course-platform with log-in	8 weeks, daily practice of 30-45 min encouraged, 6 or 7 days a week	60% completed first week, 39% completed the full mindfulness program, all but one participant found the program to some extent useful, most found the program challenging
		LT: 8 weekly modules, audio files 15-30 min exercises (formal exercises from MBSR)		
		SI: few pages of text about theoretical foundations of mindfulness in each module; possibility to phone call or e-mail study coordinators		
		PTP: informal practice exercises (e.g. awareness of routine activities)		
		JIT: weekly reminder that new module is available; additional reminders if no log-in for 14 days or weekly assessment not responded		

Lindsay et al. (2018), USA	+	Smartphone-based intervention "Mindfulness Monitoring and Acceptance"<br>LT: 20 min guided audio lesson (body relaxation, mentally welcoming of experiences, gentle labeling, equanimity)<br>SI: 5-min introductory video; Phone call on day 3 and day 9 to answer training-specific questions; possibility to phone call or text study coordinators to ask questions<br>PTP: brief unguided homework practice daily (3-10 min)<br>JIT: standardized study reminder texts and phone calls	2 weeks, 14 lessons, one lesson each day	treatment adherence 96%
Lyzwinski et al. (2019), Australia	++	Mindfulness app<br>LT: formal practice audios (e.g. breathing exercises)<br>SI: supplementary document on how to use the app, written and video lectures, practical tips and advice for mindful lifestyle<br>PTP: journal for formal and informal practice; games<br>JIT: push notifications during eating times	11 weeks, self-paced	14% reported that they reviewed all content, 23% engaged with the app very seldom, 94% liked the app and found it acceptable
Ma et al. (2018), China	+	Self-direct mindfulness based intervention<br>LT: weekly 40 min audio formal mindfulness practices (e.g. body scan, breating space, mindful sitting, …)<br>SI: mindfulness related reading material<br>PTP: cognitive therapy elements (e.g. how to recognize the thoughts related to our experience and take a different and wider perspective to experience)<br>JIT: weekly reminder	8 weeks, new material sent each week, self-paced	32 participants submitted weekly report less than five times, 15 completers

Nguyen-Feng et al. (2017), USA	+	Internet-based mindfulness only intervention, delivered via online course management system<br>LT: downloadable guided meditations focusing e.g. on breath, physical sensations, thoughts<br>SI: psychoeducational video in the beginning, short mindfulness logs (brief description of experiences) after each meditation<br>PTP: -<br>JIT: e-mail when it was time to complete tasks, reminder e-mail at the end of each week	4 weeks, one or two sessions per week	66.4% completed posttest
Querstret et al. (2018), UK	++	online mindfulness-based cognitive therapy website https://www.bemindfulonline.com/<br>LT: 10 interactive video sessions with formal meditation exercises (audios 20-30 min) and informal mindfulness techniques<br>SI: 3-min introductory video each week (is necessary to access formal practice audios), supplemental embedded content on web-page; materials from previous weeks is accessible; events diary in week 2; difficult thoughts check-list in week 3; reflection on what was learned at the end of week 4<br>PTP: informal mindfulness techniques for daily practice<br>JIT: reminder e-mail after one week of inactivity	approximately 4 weeks, self-paced, at least complete one formal and informal exercise per week	75% of participants completed the intervention

Shore et al. (2018), UK	++	"Learning mindfulness online", university virtual learning facility<br><br>LT: 10 min of daily guided mindfulness practice (audio track)<br><br>SI: welcome page with overview of topics; daily practice FAQ; Daily Journal; Information about study, help and assistance<br><br>PTP: reminder emails contained suggestions on ways in which mindfulness could be brought into everyday life (mindful eating, mindful walking, …)<br><br>JIT: standardized reminder emails at three-day intervals	2 weeks, daily practice	52% of participants completed the intervention, mean number of self-reported days of practice M = 11.8 days
van Emmerik et al. (2018), Netherlands	++	VGZ Mindfulness Coach app<br><br>LT: 40 audio mindfulness exercises (meditation, visualization, body scan, attention, mantra, yoga); optional 5-week program with 25 preselected audio exercises<br><br>SI: background information about mindfulness; information on how to use the app (filters for length, aim and setting)<br><br>PTP: ?, there are short exercises (e.g. 3 minutes) for other settings (e.g. public transport) in the app, but it is not clear whether these are instructions for informal practice or just other formal exercises<br><br>JIT: option to let exercises appear in the user's agenda app; weekly reminders in the (optional) 5-week program	8 weeks, self-paced	42% completed posttest, 26% completed follow-up, in general high satisfaction with the app among completers, average use of 3.64 weeks

Wahbeh & Oken (2016), USA	o	Internet Mindfulness Meditation Intervention	6 weeks	70.5% completed all study activities, 8 participants completed all 6 web-based lessons, intervention was higher rated on Client Satisfaction Questionnaire than control
		LT: weekly 1 hour web-based video training sessions with multiple lessons (6-10), formal audio meditation practice each day (body scan, sitting meditation, …)		
		SI: sessions including didactic instruction and discussion on topic (stress, meditation, …), problem-solving techniques regarding success and difficulty in practicing mindfulness; after each video questions about the video content; review of the previous session in beginning; summary of current session in the end		
		PTP: daily home practice guided meditations between sessions (mindful during daily activities)		
		JIT: -		
Yang et al. (2019), USA	++	"Headspace"	30 days	60% used the app at least once during 30 days period, 74% of those continued use in post-intervention period
		LT: 10-20 min guided audio meditations (45 sessions)		
		SI: probably there is some, but it is not described; log of the number of minutes that the app was used		
		PTP: probably there is some, but it is not described		
		JIT: probably there is some, but it is not described		

ER … effectiveness rating
JIT … just-in-time information
LT … learning task
PTP … part-task-practice
SI … supportive information
